# Pathway Evidence of How Musical Perception Predicts Word-Level Reading Ability in Children with Reading Difficulties

**DOI:** 10.1371/journal.pone.0084375

**Published:** 2013-12-17

**Authors:** Hugo Cogo-Moreira, Clara Regina Brandão de Ávila, George B. Ploubidis, Jair de Jesus Mari

**Affiliations:** 1 Department of Psychiatry and Medical Psychology, Federal University of São Paulo, Sao Paulo, Sao Paulo, Brazil; 2 Department of Hearing and Speech Pathology, Federal University of Sao Paulo, Sao Paulo, Sao Paulo, Brazil; 3 Department of Population Health, London School of Hygiene and Tropical Medicine, London, United Kingdom; University of Pécs Medical School, Hungary

## Abstract

**Objective:**

To investigate whether specific domains of musical perception (temporal and melodic domains) predict the word-level reading skills of eight- to ten-year-old children (n = 235) with reading difficulties, normal quotient of intelligence, and no previous exposure to music education classes.

**Method:**

A general-specific solution of the Montreal Battery of Evaluation of Amusia (MBEA), which underlies a musical perception construct and is constituted by three latent factors (the general, temporal, and the melodic domain), was regressed on word-level reading skills (rate of correct isolated words/non-words read per minute).

**Results:**

General and melodic latent domains predicted word-level reading skills.

## Introduction

Associations between musical ability and language skills have been studied over the past twenty years. Studies have found that musical ability may facilitate the acquisition of a second language, as it predicts phonological ability (both receptive and productive) even when controlling for other factors [1]. Musical metrical perception, as one domain of musical perception, has an organisational function in the phonology of language and phonological learning via speech prosody. For example, it enables the accurate segmentation of syllables and words from the speech stream [2], which is likely critical for phonological development and, consequently, the development of literacy [3].

Musical training has been found to facilitate the processing of lexical stress [4], verbal memory [5], verbal intelligence, and executive function [6], as well as the neural encoding of speech [7] latter caused by experience-dependent neural plasticity in the brain stem [8]. Indeed, other studies have provided further evidence of the correlation between the enhancement of neural encoding of spoken syllables in the auditory brain stem and the amount of musical training [9,10]. Moreover, musical expertise has been shown to influence prosody [11,12] and perceptual processing as well as the categorisation of linguistic contrasts in a foreign language [13]. 

On the basis of these findings, it has been hypothesised that musical intervention for children with reading difficulties would be helpful because musical aptitude and literacy are both related to the extent of subcortical adaptation to regularities in on-going speech and to auditory working memory and attention. Therefore, similar brain mechanisms underlie reading and musical abilities [14,15]. The “OPERA” hypothesis [15,16] specifies why such benefits of musical training for linguistic abilities occur, since these benefits are driven by adaptive plasticity in speech-processing networks. 

One study, however, has indicated that there is little agreement on the particular elements of musical perception that may correlate with reading difficulties among school-aged children [17]. Auditory analysis skills used in the processing of language, such as blending and segmenting sounds, are similar to the skills necessary for musical perception, such as rhythmic, melodic, and harmonic discrimination, despite the differences in the nature of the stimulus (one musical and the other linguistic) [18]. Therefore, it is reasonable to hypothesise that, because early reading skills are closely related to the skills for processing auditory components of speech, the different elements of musical perception (timbre, rhythm, harmony, and pitch) may also be associated with reading development [19].

A systematic review was conducted to evaluate the effectiveness of music education on reading skills among children and adolescents with dyslexia. The review employed a sensitive search using *dyslexia* (a specific learning disability) and general descriptive terms such as *reading difficulties* and *reading problems* to identify randomised clinical trials in which music education was used to improve the reading skills of children and adolescents. Among more than 700 citations without language restrictions, no randomised, controlled clinical trials were published until June of 2012 [20]. However, recently, a pragmatic-cluster, randomised clinical trial (RCT) conducted in ten public schools in São Paulo found promising results (intervention was offered over 5 months, 3 times per week, 50 minutes per class) among children from eight to ten years old with reading difficulties. Specifically, considering the complier average causal effect analysis, in which complier children were compared with non-complier children, complier children showed an improvement in the rate of correct words read per minute and phonological awareness (i.e., word-level reading skills) and in the academic achievement in Portuguese and math grades throughout the school year [21]. In another study, a pseudo-randomised clinical trial, among children without previous reading difficulties, researchers observed enhanced reading and pitch discrimination abilities in speech in a music training intervention [22]. The explanation for the causal paths to reading development via musical training may be referred to as “transfer” [23,24]; the connection between musical learning and improved reading skills is a “far transfer” because musical learning is not directly related to reading or academic achievement in Portuguese or math. Musical training is based on teaching and constant practice of non-verbal structures such as classical sheet music and contact with elements that are present in musical phenomena (e.g., rhythm, melody, metre, and so on), whereas reading is related to verbal language. An example of “near transfer” is the acceleration of the development of harmony perception in four- and five-year-olds due to musical training [25]. An important question to ask about the promising results of music education (i.e., musical learning) on the reading skills among children who are poor and not-so-good readers in the first (above-cited) randomised clinical trial of this intervention is whether the improvement in reading and academic achievement was *caused by* the improvement in musical perception. The authors only collected measures related to musical perception skills at baseline. Therefore, it was not possible to determine how and why music education (and, thus, a potential improvement in musical perception skills) might improve reading and academic performance. The pragmatic randomised clinical trial was designed, as the name indicates, to reflect the heterogeneity of children with reading difficulties. It was not concerned with a specific learning diagnosis and, therefore, focused on the effectiveness (not efficacy) of music education for word-level reading skills; in other words, it took into account a more heterogeneous population with reading difficulties despite specific learning language disorders (for major considerations regarding the nature of pragmatic RCT, see Hotopf’s work [26]).

The purpose of the current study is to test whether musical perceptions and their specific latent domains predict word-level reading skills (e.g., decoding of words and non-words) using a pathway analysis with latent factors. 

## Methods

This study was conducted using the baseline measurements and sample from a randomised clinical trial of the effectiveness of music education for improving the reading skills and academic achievement of children with poor reading skills [21]. 

### Participants

The sample comprised 235 children from eight to ten years old (females = 38.3%; males = 61.2%), with an average age of 9.15 years (SD = .05), with reading difficulties from ten public schools in impoverished neighbourhoods on the outskirts of the city of São Paulo, Brazil. Forty-eight teachers from the ten schools were asked to complete the Scale of Assessment of Reading Competence of Students by the Teacher (EACOL), with the following instructions: “. . . for the children in your class with reading ability below the mean for the corresponding grade, please fill out the EACOL”. The EACOL contains 27 dichotomous items that evaluate the following two domains of elementary children’s reading competencies: reading aloud (17 items; related mostly to automatised word recognition and decoding skills) and silent reading ability (10 items; related mostly to comprehension). The EACOL distinguishes three latent groups of readers (good reader, not-so-good reader, and poor reader), showing discriminant and concurrent validity [27]. Scores can range from -29 to +29 points and are interpreted as follows: poor reader (< -14.5), not-so-good reader (from -14.5 to 14.5), and good reader (> 14.5). Of the 235 children, 82.8% were classified as poor readers, and 17.2% as not-so-good readers. Because the RCT’s primary aim was to evaluate the effectiveness of music education among children who have reading difficulties, only the latent classes of the two below-average reading competencies were considered.

Lastly, other important features of this sample are as follows:

• The children’s non-verbal intellectual ability, measured by Raven’s coloured progressive matrices [28], was above the 25th percentile; according to Brazilian norms to Raven’s coloured matrices, children who are in the 25th percentile are defined as definitely below the average, while those are considered intellectually defective who are at the 5th percentile or lower. Moreover, at the time when this research was conducted (2011), we used as a diagnostic criterion for mental retardation the DSM-IV, which determines, among other things, that “the essential feature of mental retardation is significantly subaverage general intelligence.” It is determined by a standardized, individually administered intelligence test in which overall full-scale IQ score and verbal/performance-scale IQ scores are found to be at least two standard deviations below the mean, with the standard error of measurement accounted for. Statistically, it is known that, under the normal curve, two standard deviations below the mean is equivalent to one percentile lower than 5 (for major clarification see Urbina, 2004 [29], Figure 2.2). Furthermore, in relation to the Brazilian educational system, it is noteworthy that children with mental retardation generally study in specialized schools. In recent years, efforts have been conducted to ensure that children who have mental retardation are included in the normal education system, which is not done unless they undergo evaluations that indicate their current level of development. Therefore, the adopted cut-off was conservative, guaranteeing that children with mental retardation were excluded from the sampling.
• They did not receive any regular language-speech therapy or music classes (such as private music classes in a conservatory or other school of music, e.g., in a social project involving musical learning).

Information on these two features was obtained from the parents who provided consent for their children to participate in this study. The letter to parents requesting such consent explained the study’s aims, procedures, and measurements, avoiding technical scientific vocabulary. We also requested the parents’ written informed consent, which was approved by the Ethical Committee from the Federal University of São Paulo (CEP0433/10), for their children’s participation. Only the children whose parents gave written consent were included in the study. All written informed consents were stored at the Department of Psychiatry at the Federal University of São Paulo. This study was approved by the Ethical Committee from the Federal University of São Paulo.

The variables used to build the model to test whether musical perception and its specific latent domains predict word-level reading skills are described below.

### Covariates assessment

 In order to control for the likely relevant effects of covariates related to reading in text, the following assessments were conducted:

• The Simplified Auditory Processing Test (SAPT) [30] was conducted by a hearing and speech pathologist. The following auditory abilities were tested: sound localisation in five directions and verbal and non-verbal sequential memory, corresponding to the processes of localisation and temporal time ordering. The children were classified as having or not having problems in central auditory processing.
• For Intelligence Quotient (IQ), the complete Brazilian Portuguese validation of the Wechsler Intelligence Scale for Children, Third Edition (WISC-III), was used and administered by a trained psychologist [31].
• To measure the ability to analyse metaphonological skills, we used the Test of Phonological Awareness [32]. The test is composed of ten subtests, each consisting of four items that test the following: synthesis, segmentation, syllabic, and phonemic manipulation and transposition, and rhyme and alliteration.
• The visual acuity of the children (age-appropriate) under conditions of monocular viewing was tested by an ophthalmology technician using Snellen’s chart. The children were classified as either having visual alterations or not.

### Word-level reading skills assessment

#### Decoding

 Variables related to word-level reading skills were collected to build the model. Such skills were chosen based on the Simple View of Reading [33], which advocates that word reading and language comprehension are the primary component skills, and that limitation in either skill contributes to compromised reading comprehension, which is the ultimate goal of reading instruction [34]. These word-level skills were:

• accuracy of word task (rate of correct words read per minute)
• accuracy of non-word task (rate of correct non-words read per minute)

The lists used to assess skills had a total of 88 words and 88 non-words. The lists contained both high- and low-frequency words, words with bi-directional regularity (regular and irregular words according to grapheme-phoneme/phoneme-grapheme correspondence), and words of various lengths (short, medium, and long words in terms of the number of letters). The non-words were built with identical orthographic Brazilian Portuguese structures and had the identical length of stimuli used in the list of words. The children were asked to read aloud the words and non-words, and the time spent reading was computed. Segmentation and prolongation in the children’s reading were considered faults. 

The correlation between word and non-word accuracy tasks was high (r =.92, p < .001); the tasks showed a moderately positive correlation with the Phonological Awareness Test [32] (r _accuracy of word_ = .40, p < .001 and r _accuracy of non-word_ = .37, p < .001). As expected, the general Intelligence Quotient (IQ) was poorly related to word accuracy (r = .168, p = .01) and not correlated with non-word accuracy (r = .01, p = .131). For more detailed information about the validity of word and non-word tasks, see 27.

#### Fluency in text

Accuracy of text (rate of correctly read words per minute) was obtained with consideration for the age of the child. For the eight-year-old children, we used *The Tortoise and the Leopard* [35]; for the nine-year-olds, we used *The Nut Veterinarian* [36]; and for the 10-year-olds, we used *The Owl and the Eagle* [36]. The children’s reading was recorded for post-analysis of accuracy. The baseline assessments of accuracy of text (rate of correct words read per minute) was highly correlated with the accuracy of words (r = .916; p < .001) and the accuracy of non-words (r = .873; p < .001).

#### Musical perception

The Montreal Battery for Evaluation of Amusia (MBEA), which consists of six subtests (scale, contour, interval, rhythm, metre, and melody memory), was used to evaluate the mechanisms that underlie musical perception. The MBEA does not rely on the memory of familiar melodies and was developed considering the cognitive theories of musical perception and neuropsychological evidence [37]. Children are given 10 minutes to work on each of the six subtests. The subtests are composed of 30 to 31 trials, which are preceded by at least two examples, with feedback and question answering allowed. No feedback is provided during the test. The participants listened to two melodies and indicated whether these melodies were the same or different. The Portuguese version of the MBEA was validated for the Brazilian social and cultural context [38]. For this study, the melody memory subtest (the last MBEA subtest) was not used because the majority of children asked to stop the examination during the first days of baseline assessment due to fatigue. Therefore, for ethical reasons, only five subtests from the MBEA were used.

### Statistical analysis

#### Confirmatory factor analysis of the MBEA: The general-specific model

Instead of using the summed five subtest scores to compose the musical perception ability, as it is usually done (for example, Cuddy et al. [39]), pitch-based tests (which are called here melodic domains, constituted of scale, contour, and interval subtests), temporal-based tests (constituted of metric and rhythm subtests), and musical perception (constituted of the five subtests together) were modelled. In order to achieve this aim, a confirmatory factor analysis (CFA) was run to evaluate the impact of melodic, temporal, and musical perception domains—as latent factors as hypothesized in previous studies [37,40]—on word-level reading skills. In other words, the three domains are treated as unobservable variables, as musical perception is a general factor and melodic and temporal domains specific factors. 

The correlations between the general-specific factor (also called bi-factor model) and the specific factors (i.e., melodic and temporal domains) and between the specific factors and the general factor (i.e., musical perception) were fixed at zero. The general factor is loaded (explained by) all five MBEA subtests; specific refers to two latent factors (melodic and temporal) that account for the association between musical perception indicators and the specific dimensions/factors. The Comparative Fit Index (CFI), the Tucker Lewis Index (TLI), and the Root Mean Square Error of Approximation (RMSEA) were used to evaluate the model’s fit. The CFI refers to the discrepancy function adjusted for sample size. The TLI assesses the incremental fit of a model compared to a null model. Both range from 0 to 1, and an acceptable model fit is indicated by a CFI and TLI value of .95 or greater. The RMSEA is related to residuals in the model ranging from 0 to 1, and an acceptable model fit is indicated by an RMSEA value of .06 or less. The Standardised Root Mean Square Residual (SRMR) is an absolute measure of fit and is defined as the standardised difference between the predicted correlation and the observed correlation; a value less than .08 is generally considered a good fit. These indices of fit and a chi-squared goodness of fit test were used to assess the model fit, as suggested by existing guidelines [41]. 

The CFA, as a first step in developing an integrative model, was utilised to create latent domains underlying the MBEA’s musical perception construct. Latent domains present opportunities to understand how temporal and melodic domains (as latent domains) could affect word-level reading skills better than each of the MBEA’s subtests separately or simply by adding the scores from the subtests. 

#### Integrative model—reading skills and musical perception abilities

The general-specific factor model of musical perception (based on theoretical and statistical considerations) was regressed on reading variables and confounders. The rationale for the causality concatenation among the variables in the integrative model was based on the following assumptions: (a) musical perception influences word-level reading skills (i.e., word and non-word accuracy); (b) these skills influence fluency in text (i.e., rate of correct words read per minute); and (c) confounders impact fluency in text. Therefore, there is concatenation beginning with musical perception, passing through word-level skills to fluency in text. 

The accuracies of words and non-words were treated as censored from below (that is, having a floor effect) because an excess-zero issue may cause significant correlation coefficient results and false correlation findings in the proposed structural equation. Because the sample consisted of children with reading difficulties, some children scored zero correct read words and non-words per minute, generating floor effects in both word-level reading measures. The maximum likelihood estimator was used to perform the integrative model (reading variables, confounders, and musical perception). To account for the non-independence of observations (i.e., children nested in schools, which generated a multilevel structure), standard errors and chi-square test of model fit took account such non-independence of observations due to the cluster sampling were computed; the implementation of these methods in Mplus is discussed in Asparouhov and Muthén (2005, 2006) [42,43]. Likely problems related to non-convergence due to negative residual variances and correlations greater than one caused by dependence in the data set were inspected. All continuous variables and covariates were tested for univariate normality based on skewness and kurtosis tests. 

The structural equation modelling (CFA and path analysis) was conducted via Mplus 7 [44].

## Results

### Sample description

The current sample included 235 children (38.29% females; 61.71% males), of whom 37.87% had problems in auditory processing and 34.04% had visual acuity problems. The average age was 9.15 (SD = .05). The mean number of children in each school was 23. Table 1 displays means and standard deviations (SD), kurtosis, skewness, and a univariate normality test (skewness and kurtosis normality test) of reading measures and other collected continuous variables. 

**Table 1:** Mean, standard deviation (SD), kurtosis, skewness, and univariate normality test (skewness and kurtosis normality test) for all continuous (observable and latent) variables.

Variables	Mean	SD	Kurtosis	Skewness	S-K normality test (p-value)
Age	9.19	0.81	2.27	0.28	<0.001
IQ total	92.40	13.92	2.89	0.13	0.68
Phonological awareness	24.88	5.12	3.44	-0.17	0.2
Accuracy of word	10.34	12.95	4.91	1.53	<0.001
Accuracy of non-word	5.53	7.44	5.44	1.72	<0.001
In-text accuracy	26.17	21.36	3.76	1.03	<0.001
General musical perception Factor score	0.00*	1.09	2.63	0.30	0.08
Melodic factor score	0.00*	0.74	3.31	0.19	0.25
Temporal factor score	0.00*	1.60	3.11	-0.11	0.67

Abbreviations: SD = standard deviation; S-K = skewness and kurtosis

^*^ Differences in the mean of factor scores are observable only in the fifth value following the period. To avoid scientific notion (i.e., 4E-5), we opted to present the mean of factor loadings as zero.

### The musical perception model fit

We ran the musical perception model isolated from the other measures; two children were absent at the time of evaluation. Three models of the MBEA’s underlying construct were tested via CFA, as follows: a general-specific model, a second-order model, and a general model. 

The general-specific model displayed the following indices: CFI = 1.0, TLI = 1.019, RMSEA = 0, and SRMR = .012. For each domain, factor scores were obtained and were based on the regression methods (also known as the maximum posterior estimator, see 45). To avoid the sum of subtests, we computed the factor scores, resulting in a measure of latent domains. The factor scores for each of the domains were normally distributed, with a mean at zero and standard deviation close to one (Table 1).

### Musical perception, reading, and confounders—an integrative model

**Figure 1:**
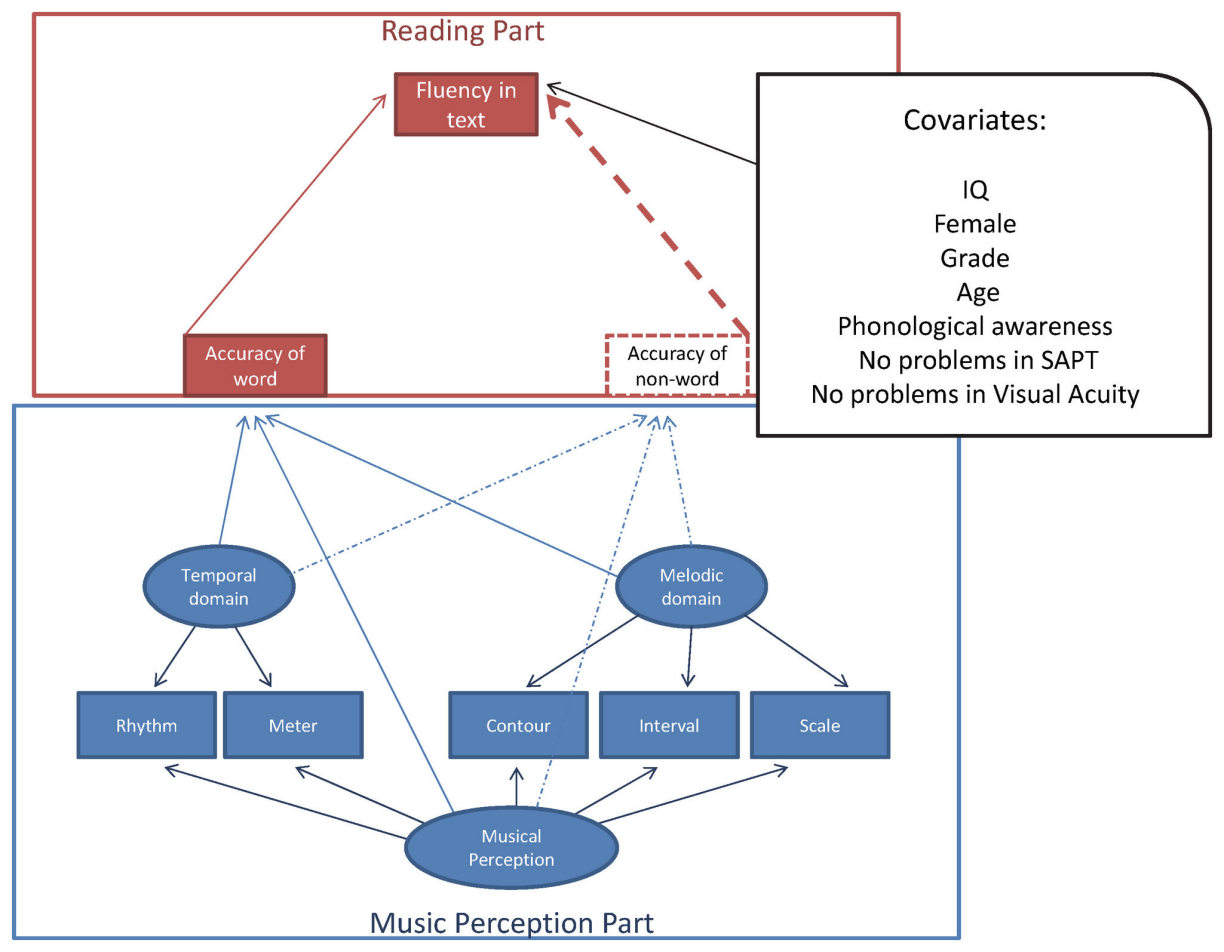
Integrative model with convergence problems.

The first integrative model, in which the general-specific model was regressed on word and non-word accuracy and confounders, is shown in Figure 1 (dashed and solid lines and squares).

The model returned a non-positive residual covariance matrix. No negative variance, residual variance, or even correlation of one or greater was observed. Dependency among some variables may have caused the convergence problems due to a high correlation among the variables. Specifically, word and non-word accuracy may have generated this convergence problem, and, therefore, non-word reading was excluded from the model. Because a convergence problem is an inadmissible solution, we present the diagram only to provide the hypothesised model, but related standardised coefficients (and their statistical significances) are not presented. We excluded the non-reading accuracy route (dashed lines) from the model because, when children develop reading fluency, they abandon the phonological strategy, which is more related to non-word reading skills and rely on the graphemic encoding procedure.

**Figure 2:**
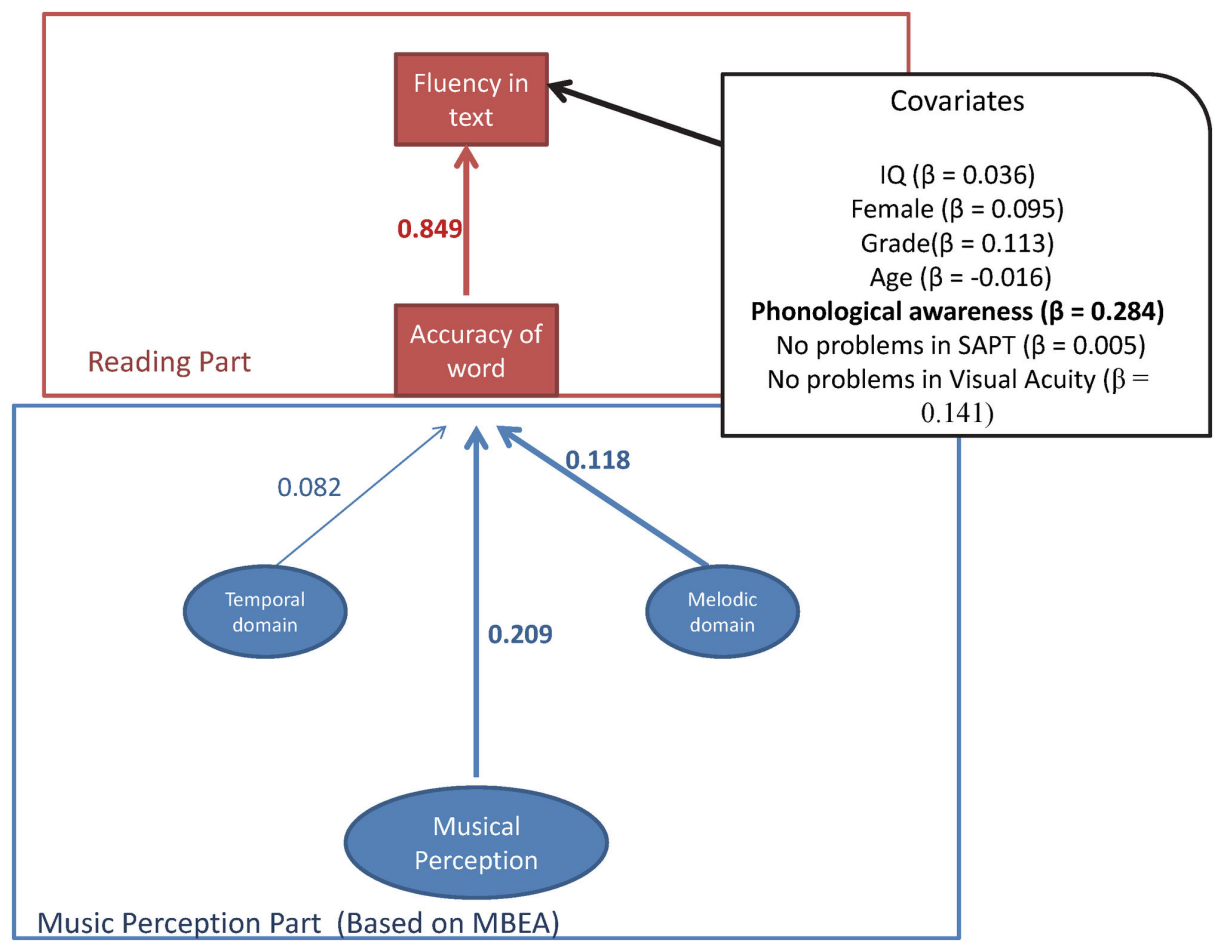
Integrative model with standardised coefficients.

Due to the non-positive matrix, a second model (without the non-word reading achievement) was tested (Figure 2), in which the general factor of musical perception (b = 2.57, p = .014) and a specific part (the melodic domain [b = 2.105, p = .035]) were predictors of word accuracy. Thick lines and bold variable descriptions in Figure 2 indicate statistically significant trajectories, and presented values are the standardised coefficients. 

Regarding the covariates, phonological awareness (b = 1.318, p < .001) was a statistically significant predictor of in-text accuracy (fluency in text). All of the above regression coefficient values are non-standardised (called “b”). In Figure 2, they are standardised. Concerning the 37.87% with auditory problems, a Brazilian cross-sectional study (probabilistic sampling) [46] conducted in the Minas Gerais state showed, using the same assessment of SAPT protocol, a prevalence of 16.30% (the range for the true population proportion, taking 95% confidence interval, was 11.94% to 20.66%). Because the inclusion criterion of our study was children with reading difficulties, as a consequence it was expected higher percentage of children with auditory problems. 

## Discussion

A high correlation between word and non-word accuracy (r = .92, p < .001) suggested that both decoding measures tapped the same construct rather than two distinguishable constructs. Indeed, because the children in this study had weak word-reading skills, the word-reading task may have, in effect, been a non-word-reading task for them. This high correlation motivated the omission of non-word accuracy in the integrative model (dashed square and its dashed trajectories) to avoid convergence problems. 

The melodic domain was a predictor of word accuracy (model with solid lines). This dissociation involving a specific domain of musical perception may be related to the independent processing of musical (melodic and temporal) domains. The traditional neuropsychological view has treated them separately [47]; however, other authors have argued that perception, attention, and memory for pitch relations are inherently rhythmic because the perception of melody and rhythm is treated as a unified dimension [48]. 

The decision regarding the underlying structure of the MBEA as a general-specific model was theoretical, as hypothesised [37], corroborating descriptions about two parallel and independent subsystems [49].

The melodic domain (a specific part of the musical perception model) as a predictor of word accuracy may be related to the phonological skills that are required in word-level reading tasks. One study found that global pitch processing (related to the melodic domain) and reading component skills were restricted to the phonological domain, establishing a semi-partial correlation between global pitch perception tasks and non-word repetition tasks (r = .38; p < .05) and speed in reading aloud a list of non-words (r = .41; p < .05) [50]. 

Because the children in the current study had difficulties in reading, as previously mentioned, the list of words may have served as non-words, generating convergence problems when the accuracy of words and non-words were used in the same model. 

The present results were consistent with previous findings, especially with studies involving children with dyslexia; however, it is important to emphasise that the current sample was not given such a diagnosis (dyslexia). Therefore, the results may indicate the following: (1) achievement on pitch-based tasks (as a predictor) is not exclusive to children with dyslexia—it also occurs in children with reading difficulties (remembering that only word-level decoding skills were evaluated in the present study); and (2) a possible explanation of how and where musical learning might improve the development of word-level reading skills among children with reading difficulties. However, there is a methodological limitation in the latter perspective. It is based on a cross-sectional point of view because we only considered baseline measurements in a randomised clinical trial via structural equation modelling. Consequently, causality cannot be completely determined. A study involving children aged eight to eleven years old found a link between impaired pitch processing and abnormal phonological development in children with dyslexia, demonstrating that pitch pattern processing is an important predictor for the early diagnosis and remediation of dyslexia [51]. In contrast with these findings, another study has shown that enhancement of phonological awareness among pre-schoolers was driven by more general positive effects of the music program [52]. 

Our purposed pathway analysis was centered on word-level reading performance) and music perception; future studies should look, for example, at the same children’s arithmetical abilities, other school-related knowledge, or even comprehension reading skills (“high-level” reading skill), evaluating the plausibility of transferences due to music perception skills to other or likely correlated domains; a particularity of our sample is that children came from lower SES, which strongly per se impacts on reading performance [53] and influences the relationship between phonological awareness and reading ability [54]. Therefore, children coming from lower SES who have struggled with word-level reading most probably struggle in all or most school-related domains as well, which were not measured here. In such a context, a strong point to be addressed in our analysis regarding modeling of the effects of the selected schools from impoverished neighborhoods is the non-independence of observation (children) due to the cluster sampling were computed via a multilevel analysis. 

For children with dyslexia, the link between written language and musical perception is at the rhythmic level, not at the level of pitch, as the simple rhythm reproduction tasks in kindergarten are predictive of later reading performance [55].

Regarding the musical perception evaluation, it is important to stress that the MBEA is based on the paradigm of Western European music of the tonal period (more specifically, it considers the majority of the items to have a diatonic structure). Other paradigms underlying a musical perception model involving atonal structure and other spectra of metrics (e.g., 7:8, 12:15) and timbres (white or pink noise) were not considered but might be examined in future studies involving predictions of musical perception and its relation to reading skills. Additionally, the current study’s reading variables represented levels of decoding and, as a consequence, other reading skills (higher-level language skills related to comprehension). There is a strong body of evidence demonstrating that phonological awareness is a predictor of reading development and spelling [56,57].

Lastly, given the nature of our sample, an advantage to stress is that the sample makes it possible to infer, among children with reading difficulties, that there is pathway in common with those of other specific reading disorders, such as dyslexia. Indeed, to determine the kind of language impairment, perceptual or speech-related, from which a child is suffering would be helpful and important for understanding different underlying process relating music perception and word-reading skills in the whole spectrum of language disorders, testing the invariance-of-concept model throughout different pathologies.

## Conclusion

As a main conclusion, the MBEA’s melodic domain was a predictor of word-level decoding skills among children with reading difficulties, corroborating evidence of the dissociation of musical perception in the temporal and melodic domains.
